# Routine HIV Screening in Two Health-Care Settings — New York City and New Orleans, 2011–2013

**Published:** 2014-06-27

**Authors:** Xia Lin, Patricia M. Dietz, Vanessa Rodriguez, Deborah Lester, Paloma Hernandez, Lisa Moreno-Walton, Grant Johnson, Michelle M. Van Handel, Jacek Skarbinski, Christine L. Mattson, Dale Stratford, Lisa Belcher, Bernard M. Branson

**Affiliations:** 1 EIS officer, CDC; 2 Division of HIV/AIDS Prevention, National Center for HIV/AIDS, Viral Hepatitis, STD, and TB Prevention, CDC; 3 Urban Health Plan, Inc., New York, New York; 4 Section of Emergency Medicine, Louisiana State University Health Sciences Center

Approximately 16% of the estimated 1.1 million persons living with human immunodeficiency virus (HIV) in the United States are unaware of their infection and thus unable to benefit from effective treatment that improves health and reduces transmission risk (1,2). Since 2006, CDC has recommended that health-care providers screen for HIV all patients aged 13–64 years unless prevalence of undiagnosed HIV infection in their patients has been documented to be <0.1% (3). This report describes novel HIV screening programs at the Urban Health Plan (UHP), Inc. in New York City and the Interim Louisiana Hospital (ILH) in New Orleans. Data were provided by the two programs. UHP screened a monthly average of 986 patients for HIV during January 2011–September 2013. Of the 32,534 patients screened, 148 (0.45%) tested HIV-positive, of whom 147 (99%) received their test result and 43 (29%) were newly diagnosed. None of the 148 patients with HIV infection were previously receiving medical care, and 120 (81%) were linked to HIV medical care. The ILH emergency department (ED) and the urgent-care center (UCC) screened a monthly average of 1,323 patients from mid-March to December 2013. Of the 12,568 patients screened, 102 (0.81%) tested HIV-positive, of whom 100 (98%) received their test result, 77 (75%) were newly diagnosed, and five (5%) had acute HIV infection. Linkage to HIV medical care was successful for 67 (74%) of 91 patients not already in care. Routine HIV screening identified patients with new and previously diagnosed HIV infection and facilitated their linkage to medical care. The two HIV screening programs highlighted in this report can serve as models that could be adapted by other health-care settings.

UHP, a federally qualified health center network of eight practice sites and eight school-based health centers, serves approximately 60,000 unique patients each year. ILH, a public hospital, serves approximately 76,000 unique patients in its ED and UCC each year. Both received startup funding from Gilead Sciences’ HIV on the Frontlines of Communities in the United States (FOCUS)* program to implement routine HIV screening based on four principles: 1) institutional policy change reflecting an organization-wide commitment to routine HIV testing and diagnosis; 2) integration of HIV testing into existing clinical workflows to promote its normalization and sustainability; 3) use of electronic health records (EHR) to prompt testing, automate laboratory orders, and track performance; and 4) required staff education on best HIV testing practices and outcomes.

Before FOCUS, UHP counselors conducted risk-based, point-of-care rapid or laboratory HIV tests. With the new routine supported by FOCUS at UHP from January 2011 to September 2013, a medical assistant provides HIV information required by New York state, offers an HIV test to all patients aged 13–64 years with no documented HIV test within 12 months, and documents the offer in the EHR. The EHR prompts the health-care provider to confirm the patient’s agreement, and the health-care provider orders an HIV laboratory test. Negative test results are provided at the patient’s next visit or by letter. The program coordinator contacts patients who test positive and schedules an appointment to receive their test results and follow-up at the center that provides primary HIV medical care. The UHP commercial laboratory uses an HIV antibody assay and Western blot that detects established but not acute HIV infection, the highly infectious stage before antibodies to HIV develop that contributes disproportionately to HIV transmission (4).

Before March 2013, when support from FOCUS began, ILH conducted opt-in HIV screening with point-of-care rapid tests 70 hours a week using staff dedicated only to HIV testing and counseling. Now the EHR prompts an HIV test offer at triage to all ED and UCC patients aged ≥13 years who have had no documented HIV test within 6 months. Unless the patient declines, the HIV test is ordered and processed in the hospital laboratory 24 hours a day, 7 days a week. Test results are delivered during the same visit. Patients who test positive receive CD4+ T-lymphocyte cell count and HIV viral load tests, meet with a navigator, and are linked to local HIV care facilities. The ILH laboratory uses an HIV antigen/antibody combination assay and, if necessary, a nucleic acid test to detect acute or established HIV infection.

Each program provided data on the testing outcomes before and after the new screening programs, which were collected from EHRs (last updated in March 2014). At UHP, new diagnosis and linkage to care† were based on patient report and chart review. ILH defined a new HIV diagnosis as one not previously reported to the HIV surveillance system; linkage to care was based on chart review.

At UHP, the percentage of patients tested for HIV increased from 8% during calendar year 2010 to 56% during January 2011–September 2013. The monthly average number of patients screened increased from 188 during 2007–2010 to 986 during the routine screening period. Of the 3,358 patients screened in 2010, 19 (0.57%) tested HIV-positive, of whom three (16%) were newly diagnosed. Of the 32,534 patients screened during January 2011–September 2013, 148 (0.45%) tested HIV-positive, of whom 147 (99%) received their test result and 43 (29%) were newly diagnosed. The prevalence of newly diagnosed HIV infection was higher among males (0.25%) than females (0.08%), non-Hispanics (0.23%) than Hispanics (0.12%), and persons aged ≥31 years (0.18%–0.19%) than persons aged ≤30 years (0.08%) (Table 1). None of the 148 patients diagnosed with HIV were previously receiving medical care, and 120 (81%) were subsequently linked to HIV medical care.

At ILH, the HIV screening program increased the percentage of patients tested from 17% (ED) and 3% (UCC) during calendar year 2012 to 26% (ED) and 17% (UCC) from mid-March to December 2013. The monthly average number of patients screened increased from 821 during 2010–2012 to 1,323 in the 2013 period. Of the 11,257 patients screened in 2012, 106 (0.94%) tested HIV-positive, of whom 54 (51%) were newly diagnosed. Of the 12,568 patients screened from mid-March to December 2013, 102 (0.81%) tested HIV-positive, of whom 100 (98%) received their test result, 77 (75%) were newly diagnosed, and five (5%) had acute HIV infection. The prevalence of newly diagnosed HIV infection was higher among males (0.89%) than females (0.28%), blacks (0.63%) than whites (0.49%), Hispanics (1.00%) than non-Hispanics (0.60%), and persons aged 23–30 years (0.92%) than in age groups <23 (0.68%) and >30 years (0.32%–0.71%) (Table 2). Among the 102 patients testing HIV-positive, 91 (89%) were not previously receiving medical care; 67 (74%) of these 91 patients, including the five patients with acute HIV infection, were linked to HIV medical care.

## Discussion

The findings of both FOCUS programs demonstrate that routine HIV screening using existing clinical staff increased the numbers of patients tested and diagnosed with HIV infection. The prevalence of undiagnosed HIV infection at both programs exceeded CDC’s recommended threshold (≥0.1%) for routine screening (3), and most persons previously diagnosed with HIV infection at both programs were not receiving medical care. UHP and ILH identified patients with undiagnosed and previously diagnosed HIV infections and successfully linked the majority to HIV medical care. Active linkage is an essential element of a routine screening program to ensure that HIV-infected persons receive HIV care and services. These integrated routine HIV screening programs can serve as models for other emergency and primary health-care settings.

Several factors associated with the FOCUS principles, including supportive institutional policy changes, EHR prompts, staff education, and conventional laboratory testing for HIV, contributed to these sustainable and scalable routine HIV screening programs. Similar EHR prompts, provider training, and periodic feedback led to immediate and sustained increases in HIV testing in Veterans Healthcare Administration facilities during 2009–2011 (5). New laboratory testing methods can reduce turnaround time for test results, are more sensitive during early infection, and can detect acute HIV infections. The transition from point-of-care rapid testing to laboratory testing reduced staff time (6) and costs (7), increased feasibility to test larger numbers of patients, and allowed ILH to detect acute HIV infections. Almost all patients who tested HIV-positive received their test results. UHP received FOCUS support in the first 2 years but has continued the HIV screening program without external funding. Replication of the FOCUS model has begun; UHP staff trained five federally qualified health centers in New York City in 2013 to implement routine HIV screening.

The findings in this report are subject to at least four limitations. First, it was not possible to assess how much each factor of the new screening strategy individually contributed to the increase in screening. Second, the findings from this study might not be generalizable to other clinic settings with different HIV prevalence. Third, UHP might have underestimated HIV infections because its laboratory testing was unable to detect acute HIV infection. Finally, linkage to care might be underreported if it occurred at a different care facility.

Routine HIV screening with an active linkage element reduces the number of persons unaware of their HIV infection and links patients to medical care. These patients are then able to benefit from effective treatment to improve health and reduce transmission risk (2). The two programs highlighted in this report screened more patients for HIV by using EHR prompts, conventional laboratory testing, and provider training and feedback. Combined, these techniques identified more patients with HIV infection and linked them to care by adopting practices that other health-care settings might choose to replicate.

What is already known on this topic?

In 2006, CDC issued recommendations for routine human immunodeficiency virus (HIV) screening of adults, adolescents, and pregnant women in health-care settings. However, many clinical settings have not adopted routine screening. Routine screening promotes the linkage of HIV-infected persons into medical care. This allows them to benefit from effective treatment, which improves their health and reduces HIV transmission.

What is added by this report?

Electronic health record prompts, staff education, and shift from point-of-care rapid testing to laboratory testing were features that made routine HIV screening programs successful at the Urban Health Plan in New York City and the Interim Louisiana Hospital in New Orleans. This allowed integration of HIV screening into clinic workflow, scalability (i.e., the ability to expand the number of patients screened), and sustainability. In addition to identifying patients newly diagnosed with HIV infection, routine screening also identified patients previously diagnosed but not in care, and actively linked these patients to care.

What are the implications for public health practice?

These programs made HIV screening more scalable, and linked patients to HIV care. The design is being sustained without external support at the Urban Health Plan and is being replicated in other clinics. These two programs can serve as models that could be adapted by other health-care settings.

## Figures and Tables

**TABLE 1:** Selected characteristics of persons screened for and diagnosed with HIV infection — Urban Health Plan, New York City, January 2011–September 2013

	Screened for HIV (n = 32,534)		Diagnosed with HIV (n = 148)		Previously diagnosed with HIV (n = 105)		Newly diagnosed with HIV (n = 43)		
				
Characteristic	No.	(%)	No.	(%)	No.	(%)	No.	(%)	% of total screened
**Sex**									
Male	10,080	(31)	94	(64)	69	(66)	25	(58)	0.25
Female	22,454	(69)	54	(36)	36	(34)	18	(42)	0.08
**Race**									
White	385	(1)	1	(1)	0	—	1	(2)	0.26
Black	4,129	(13)	57	(39)	47	(45)	10	(23)	0.24
Asian	58	(<1)	0	—	0	—	0	—	—
AI/AN	18	(<1)	0	—	0	—	0	—	—
NHOPI	155	(<1)	1	(1)	1	(1)	0	—	—
Biracial or multiracial	15,998	(49)	86	(58)	54	(51)	32	(74)	0.2
Unknown*	11,791	(36)	3	(2)	3	(3)	0	—	—
**Ethnicity** †									
Hispanic	27,005	(83)	89	(60)	57	(54)	32	(74)	0.12
Non-Hispanic	4,854	(15)	56	(38)	45	(43)	11	(26)	0.23
Unknown*	675	(2)	3	(2)	3	(3)	0	—	—
**Age group (yrs)**									
13–22	7,606	(23)	9	(6)	3	(3)	6	(14)	0.08
23–30	8,358	(26)	19	(13)	12	(11)	7	(16)	0.08
31–40	7,353	(23)	28	(19)	15	(14)	13	(30)	0.18
41–50	5,240	(16)	52	(35)	42	(40)	10	(23)	0.19
≥51	3,978	(12)	40	(27)	33	(31)	7	(16)	0.18

**Abbreviations:** HIV = human immunodeficiency virus; AI/AN = American Indian/Alaska Native; NHOPI = Native Hawaiian or Other Pacific Islander.

* “Unknown” includes missing, “don’t know,” and “declined to answer.”

† Ethnicity was defined irrespective of race.

**TABLE 2:** Selected characteristics of persons screened for and diagnosed with HIV infection — Interim Louisiana Hospital, New Orleans, March 2013–December 2013

	Screened for HIV (n = 12,568)		Diagnosed with HIV (n = 102)		Previously diagnosed with HIV (n = 25)		Newly diagnosed with HIV (n = 77)		
				
Characteristic	No.	(%)	No.	(%)	No.	(%)	No.	(%)	% of total screened
**Sex**									
Male	6,883	(55)	77	(75)	16	(64)	61	(79)	0.89
Female	5,685	(45)	25	(25)	9	(36)	16	(21)	0.28
**Race**									
White	2,666	(21)	18	(18)	5	(20)	13	(17)	0.49
Black	8,828	(70)	74	(73)	18	(72)	56	(73)	0.63
Asian	98	(1)	0	—	0	—	0	—	—
AI/AN	12	(<1)	0	—	0	—	0	—	—
NHOPI	8	(<1)	0	—	0	—	0	—	—
Biracial or multiracial	824	(7)	10	(10)	2	(8)	8	(10)	0.97
Unknown*	132	(1)	0	—	0	—	0	—	—
**Ethnicity** †									
Hispanic	697	(6)	10	(10)	3	(12)	7	(9)	1.00
Non-Hispanic	11,675	(93)	92	(90)	22	(88)	70	(91)	0.60
Unknown*	196	(2)	0	—	0	—	0	—	—
**Age group (yrs)**									
13–22	1,031	(8)	7	(7)	0	—	7	(9)	0.68
23–30	2,386	(19)	25	(25)	3	(12)	22	(29)	0.92
31–40	2,552	(20)	23	(23)	5	(20)	18	(23)	0.71
41–50	2,795	(22)	29	(28)	11	(44)	18	(23)	0.64
≥51	3,804	(30)	18	(18)	6	(24)	12	(16)	0.32

**Abbreviations:** HIV = human immunodeficiency virus; AI/AN = American Indian/Alaska Native; NHOPI = Native Hawaiian or Other Pacific Islander.

* “Unknown” includes missing, “don’t know,” and “declined to answer.”

† Ethnicity was defined irrespective of race.

## Footnotes

* FOCUS supports routine HIV screening programs with partners at 65 community health centers and 54 hospitals in 12 cities that account for 45% of persons of living with HIV/acquired immunodeficiency syndrome (AIDS) in the United States. Additional information is available at http://www.gilead.com/responsibility/hiv-focus-program.

† Linkage to care was defined as attendance at first medical appointment within 1 month of diagnosis.
